# Phylogenetic and Diversity Analysis of *Dactylis glomerata* Subspecies Using SSR and IT-ISJ Markers

**DOI:** 10.3390/molecules21111459

**Published:** 2016-10-31

**Authors:** Defei Yan, Xinxin Zhao, Yajuan Cheng, Xiao Ma, Linkai Huang, Xinquan Zhang

**Affiliations:** Department of Grassland Science, Animal Science and Technology College, Sichuan Agricultural University, Chengdu 611130, China; yandfoo001l@163.com (D.Y.); zxx19890727@163.com (X.Z.); chengyajuan_sau@hotmail.com (Y.C.); maroar@126.com (X.M.)

**Keywords:** *Dactylis*, subspecies, SSR, IT-ISJ, genetic diversity, phylogenetic analysis

## Abstract

The genus *Dactylis*, an important forage crop, has a wide geographical distribution in temperate regions. While this genus is thought to include a single species, *Dactylis glomerata*, this species encompasses many subspecies whose relationships have not been fully characterized. In this study, the genetic diversity and phylogenetic relationships of nine representative *Dactylis* subspecies were examined using SSR and IT-ISJ markers. In total, 21 pairs of SSR primers and 15 pairs of IT-ISJ primers were used to amplify 295 polymorphic bands with polymorphic rates of 100%. The average polymorphic information contents (PICs) of SSR and IT-ISJ markers were 0.909 and 0.780, respectively. The combined data of the two markers indicated a high level of genetic diversity among the nine *D. glomerata* subspecies, with a Nei’s gene diversity index value of 0.283 and Shannon’s diversity of 0.448. Preliminarily phylogenetic analysis results revealed that the 20 accessions could be divided into three groups (A, B, C). Furthermore, they could be divided into five clusters, which is similar to the structure analysis with K = 5. Phylogenetic placement in these three groups may be related to the distribution ranges and the climate types of the subspecies in each group. Group A contained eight accessions of four subspecies, originating from the west Mediterranean, while Group B contained seven accessions of three subspecies, originating from the east Mediterranean.

## 1. Introduction

The genus *Dactylis* L. comprises wind-pollinated and out-crossing cool-season perennial grasses belonging to the grass tribe Poeae, the subfamily Pooideae, and the family Poaceae [1,2]. This genus includes only one species, *Dactylis glomerata* L. [3], which was interpreted as monotypic, consisting of a single diverse species complex [4,5] and referred to as orchardgrass or cocksfoot grass. *Dactylis* is native to the northern hemisphere and can be found throughout Europe, temperate and tropical Asia, North Africa, and the Canary Islands [6,7,8]. It has been an important forage grass for more than 100 years in almost all temperate regions of the world.

According to chromosome number, *Dactylis* can be either diploid (2*n* = 14), tetraploid (2*n* = 28), or, in rare instances, hexaploid (2*n* = 42). Almost all diploids have restricted ranges and occur in particular regions, and together they account for about 5% of all wild *Dactylis* [9]. Tetraploids, in contrast, are distributed continuously across temperate Europe, the Middle East, West and Central Asia, and North Africa [10]. In many localities the two types are sympatric [11], hexaploids exist across limited ranges in Libya and western Egypt [11,12,13,14].

It is clear that there is a large and continuous range of variation in the genus *Dactylis*, making it difficult to validate any proposed species within the genus. Most subspecies were acknowledged by the early 1930s [15,16,17,18]. Though many researchers have studied the classification of the genus using cytology, chromosomes, or genetics, taxonomic interpretations of the genus concern distinct knowledge of its natural range, and there is no modern taxonomic treatment for interpreting all subspecies on a unified standard [4]. The taxonomist Domin, who is thought to be the first researcher to interpret taxa in the genus using herbaria and field studies, has recognized one species, *Dactylis glomerata* L., and eight subspecies [19]. According to even earlier reports [5,20], there were 17 diploid subspecies and three tetraploid subspecies. Still other researchers [21] have suggested 14 diploid subspecies and three major tetraploid subspecies, and in the summary of Lumaret [10], the genus was classified into 15 distinct diploid subspecies, three major tetraploid subspecies, and several minor tetraploid subspecies. Stewart and Ellison [22] divided the genus into 17 diploid subspecies and six tetraploid subspecies. The subspecies *santai* and “castellata” were described collectively, due to difficulties in differentiating them via flavonoid phytochemistry, enzyme, and morphology data [10]. However in the later report of Stewart and Ellison [4], in the diploid subspecies numbered 18, *santai* and “castellata” had been regarded as individual subspecies, since “castellata” was often treated as one subspecies since 1969 [9]. Consequently, in our study, we have investigated the collected subspecies, and provided a basis for further classification of these subspecies.

With the development of advances in biotechnology, DNA molecular markers have been used in the phylogenetic analysis of plants since the 1980s. Because DNA molecular markers are not affected by environment or gene expression but reflect the status of the entire genome, they offer some advantages over traditional phenotypic characters. They can be highly polymorphic while also being genetically stable, and so they are regarded as reliable tools for phylogenetic analysis. Simple sequence repeat (SSR) markers are tandem DNA repeats several nucleotides long that can be found in most eukaryotic genomes [23]. Variations in the number of repeats and in the repeats themselves provide polymorphic information at a gene locus. SSRs are considered to be the genetic markers that provide the greatest amount of genetic information [24,25,26], generally displaying high levels of polymorphism [27,28]. Therefore, SSR markers have been used to analyze the phylogenies of many plants, including *Agropyron* by Che et al., 2015 [29], *Oryza* by Nishikawa et al., 2005 [30], *Sorghum bicolor* and *Sorghum sundanense* by Zhan et al., 2008 [31], *Glycine* by Wu et al., 2000 [32], and *Cicer reticulatum* by Sethy et al., 2006 [33]. These results suggest that SSRs are an outstanding tool for phylogenetic analysis. Moreover, SSRs were used in the construction of the *Dactylis* genetic map [34] and in analyzing genetic variation in *Dactylis* [35,36]. IT-ISJ (intron-targeted intron-exon splice junction) markers are a new type genetic marker based on the conserved sequences of intron splice junction sites, which can be amplified from intronic regions. They were designed by Zheng [37] with reference to methods developed by Weining and Langridge [38]. IT-ISJ markers present several advantages over other markers, such as lower costs and higher stability than sequence-related amplified polymorphisms, as well as high levels of polymorphism [37].

In this study, SSR and IT-ISJ markers were employed to test the nine subspecies, ranging from four different distribution ranges and three different climate types. The specific objectives were: (1) to study the genetic diversity of all nine subspecies; (2) to discuss the genetic relationship of these nine subspecies, hoping to provide more information for further study into the classification of the genus *Dactylis*.

## 2. Results

### 2.1. Polymorphism and Marker Efficiency Analysis of SSR and IT-ISJ Markers

The 21 SSR primer pairs and 15 IT-ISJ primer pairs were used to amplify DNA fragments from the 20 accessions. A total of 196 bands were obtained from SSR primers, and all of these were polymorphic (100%), with an average number of 9.333 polymorphic bands per primer pair and a range of 6–13 bands. PIC values for SSRs ranged from 0.861 to 0.962, with an average of 0.909. The 15 IT-ISJ primer combinations amplified a total of 99 bands, and all of these were also polymorphic (100%) with a range of 3–10 bands. The average number of polymorphic bands per primer pair was 6.6, and the average PIC was 0.780, with a range from 0.545 to 0.957. These data indicate that SSR and IT-ISJ primer combinations exhibit high amplification efficiency and are reliable in the discovery of polymorphisms (Table 1).

The efficiencies of SSR and IT-ISJ markers were compared using the MI parameters (Table 2). The Ibav index value for IT-ISJ markers (0.54) was higher than that of SSR markers (0.49). However, the EMR index value of SSR markers (9.33) was higher than that of IT-ISJ markers (6.6). The SSR MI value (4.58) was an order of magnitude higher than that of IT-ISJ (3.54), indicating that SSRs are highly efficient markers.

### 2.2. Genetic Diversity and AMOVA Analysis of Dactylis Subspecies

Nei’s gene diversity index within subspecies ranged from 0.135 (*D. glomerata* subsp*. lusitanica*) to 0.252 (*D. glomerata* subsp*. hispanica*), and Shannon’s diversity index ranged from 0.211 (*D. glomerata.* subsp*. lusitanica*) to 0.425 (*D. glomerata* subsp*. hispanica*). The average intra-population diversity was 0.321, and Shannon’s index for all taxa as a population was 0.448. Therefore, the proportion of intra-population diversity was 71.7%, and the proportion of inter-population diversity was 28.3% (Table 3). Additional information about genetic variation statistics and Shannon’s diversity estimation for all accessions are listed in Supplementary Table S1. This indicates a high level diversity within subspecies. The average pairwise genetic similarity coefficients of the nine subspecies ranged from 0.355 (between *D. glomerata* subsp*. lusitanica* and *D. glomerata* subsp*. himalayensis*) to 0.491 (between *D. glomerata* subsp*. himalayensis* and *D. glomerata* subsp*. glomerata*).

The results of AMOVA analysis (Table 4) revealed that 19.1% and 80.9% of the variation is included among and within subspecies, respectively, indicating that there is high variation within subspecies. The AMOVA analysis of accessions from different countries revealed high variation within countries for some subspecies, consistent with the above results.

### 2.3. Phylogenetic Analysis

**Figure 1:**
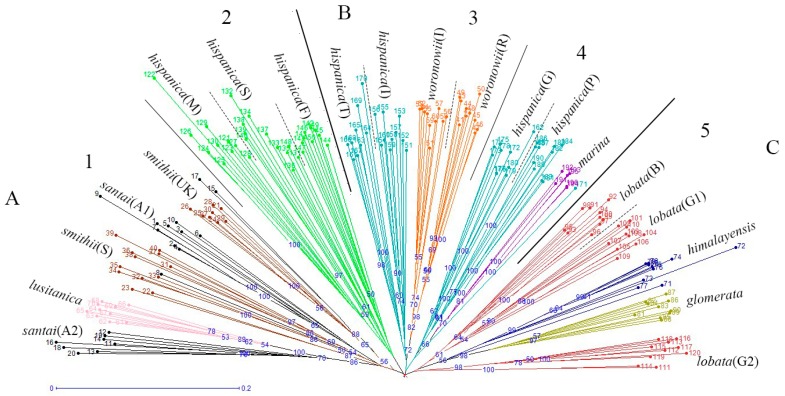
Neighbor-jioning dendrogram of 196 individuals of 20 accessions (nine *D. glomerata* subspecies), created with Darwin V5.0148. A, B and C mean the 20 accessions could be divided into three groups, and the number 1−5 indicate five clusters in accordance with the STRUCTURE analysis results when K = 5.

**Figure 2:**
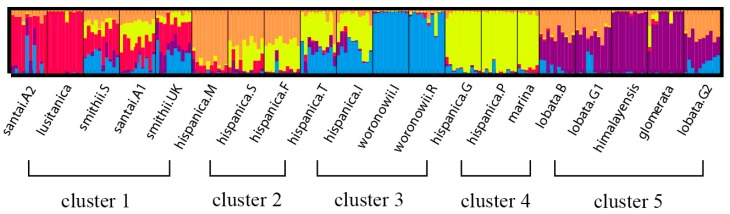
Flow chart of the STRUCTURE analysis of 196 individuals of 20 accessions (nine *D. glomerata* subspecies) at optimum K value (K = 5). The five different colors (red, orange, yellow, blue and purple) represent five genetic background, and we divided these 20 accessions into five clusters (1–5).

The neighbor joining unweighted tree (Figure 1) revealed that the individuals of the same accession could also be clustered into the same branch, and mostly the same subspecies could be clustered into the same group. The 20 accessions could clearly be divided into three groups (A, B, and C), and they could further be separated into five clusters which is similar to the structure analysis result (Figure 2) for K = 5. The results of the cluster analyses had a relationship with distribution ranges and climate distribution. The samples of Group A originated in Africa and Europe, and contained four *D. glomerata* subspecies, *santai*, *lusitanica*, *smithii*, and *hispanica*. *Santai* and *lusitanica* were climatically distributed in the Mediterranean, *smithii* was subtopical and all three subspecies could also be clustered into Cluster 1. Additionally, the three accessions of *hispanica* were also from the Mediterranean, formed Cluster 2. The samples of Group B originated in Europe or Asia-Temperate, and contained three *D. glomerata* subspecies, *hispanica, marina*, and *woronowii*, all of which originated in the Mediterranean. Furthermore, the two accessions of *hispanica* combined with *woronowii* could be assigned to Cluster 3, and the remaining two accessions of *hispanica* and the subspecies *marina* formed Cluster 4. The samples of Group C or Cluster 5 originated in Europe, and contained three *D. glomerata* subspecies, *lobata*, *himalyensis*, and *glomerata*. They originated in a Temperate climate type.

## 3. Discussion 

### 3.1. Marker Efficiency Analysis

In this study, we compared the efficiencies of two genetic markers using a marker index (MI) to estimate the utility and efficiency of each marker [39]. To our knowledge, this is the first report of a comparison of the efficiencies of the SSR and IT-ISJ genetic marker systems. The MI value for SSRs was higher than that for IT-ISJs, indicating that SSRs are more efficient and useful. The EMR component contributed highly to MI; while the Ibav values of the two marker types were similar, a higher EMR value for SSRs resulted in a higher MI value. A previous study by Powell et al. [40] compared the efficiency of SSRs with those of RFLPs (restriction fragment length polymorphism), RAPDs (random amplified polymorphic DNA), and AFLPs (amplified fragment length polymorphism). Results indicated that SSRs exhibited higher MI values than the other three genetic marker systems. Likely due to the high polymorphism rates and efficiency of the SSR marker system, it has been widely used for genetic mapping [25,41,42], phylogenetic analysis [32,43], and evaluating genetic diversity [44,45]. Although the MI for IT-ISJ was lower than that of SSR, it reached 3.54, indicating that both types of genetic markers could contribute reliable information for analyzing relationships among the nine *Dactylis* subspecies.

### 3.2. Genetic Diversity Analysis

According to Vaiman et al. [46], loci polymorphisms can be divided into three levels based on their information contents: high (PIC > 0.5), medium (0.5 > PIC > 0.25), and low (PIC < 0.25). Among the 20 accessions, all of the SSR and IT-ISJ markers exhibited PIC values > 0.5, with average PIC values of 0.909 and 0.780 for SSR and IT-ISJ markers, respectively. This indicates that both of these types of genetic markers exhibited high polymorphic information contents in our study. This result also confirms that the polymorphism content of IT-ISJ markers is similar to that of SSR markers [37]. Meanwhile, the Nei’s gene diversity index value and Shannon’s diversity index value of the total accession were 0.283 and 0.448, respectively. These values as well as the PIC values reveal that the nine *Dactylis* subspecies contain abundant genetic diversity [7,47]. *Dactylis* is a widespread genus, inhabiting many different geographical regions with different environments. In order to adapt to the environments of different geographical regions, *Dactylis* has been subject to long-term natural selection. Moreover, natural mutation and artificial selection have introduced high variation and abundant diversity in the genus.

### 3.3. Phylogenetic Analysis

The phylogenetic analysis of the 20 accessions revealed that certain phylogenetic clades are correlated with distribution range and climate type. For the most part, the subspecies of different climate type clustered into different phylogenetic branches, with the exception of a single subspecies, *smithii*, belonging to the subtropical area, while it was clustered into Group A, together with some samples belonging to Mediterranean. All three groups contained samples from Europe, indicating they have a high level of genetic diversity and variation, and this result also indicated Europe to be one of the diversity differentiation centers of *Dactylis* [48]. Generally, the origin of our samples which located in the western Mediterranean could be clustered into Group A, and those located in the eastern Mediterranean could be clustered into Group B. Several countries in the western Mediterranean are subject to Holocene climate change [49], resulting in different climates between the western Mediterranean and eastern Mediterranean. Therefore, we suspect that climate change and glaciation events may have caused genetic variation of *Dactylis* subspecies.

The two subspecies *santai* and *smithii* were clustered into Cluster 1 in Group A, showing that the two subspecies appeared closely related, which is consistent with the study of Stewart and Ellison [8], in which these two subspecies were found in the same (European and North African) clade. Subspecies *lobata*, a synonym of *D. glomerata* subsp*. aschersoniana* [22], were also in this clade (European and North African), which was slightly different from our result, clustering *lobata* together with *himalayensis* and *glomerata* and forming Cluster 5 or Group C. However, it has been reported that subspecies *lobata* and *himalayensis* have both been found in cool temperate forests at relatively high altitudes in continental climates and thus have been assigned to the same group [10]. They have similar habitats and share similar isozyme allelic patterns, flavone phytochemistries, DNA contents, and morphologies [22], indicating a close relationship between the two. Additionally, according to our study, the genetic similarity between *himalayensis* and *glomerata* was maximal at 0.491, which was also supported by the structure analysis result. The two subspecies had a very similarity genetic background, indicating that subspecies *glomerata* may have affinities with *himalayensis*, which were consistent with our phylogenetic analysis. Moreover, Stebbins and Zohary [5] suggested that subspecies *glomerata* may have evolved from the hybridization of *lobata* (*aschersoniana*) and *woronowii* and that some *glomerata* in the Alps may have evolved from the hybridization of *rechenbachii* and *lobata* (*aschersoniana*). Therefore, these three may have a very close relationship. In Group B and Cluster 4, two accessions of subspecies *hispanica* were closely related to *marina*, and structure analysis revealed them to have a very similar genetic background, which may reflect their similar distribution range and climate type. Moreover, according to the study reported by Martin Borrill et al. [9], *hispanica* is the principal subspecies found in regions with a Mediterranean climate, and *marina* Borrill can be found in Mediterranean coastal regions. *Marina* was separated from *hispanica* due to its epidermal papillae [19], but the two subspecies in Mediterranean exhibit many similar features [22]. Therefore, these two subspecies may derive from a recent common ancestor. 

## 4. Materials and Methods

### 4.1. Plant Materials

We selected 20 plant introduction (PI) accessions from 14 countries, including nine *D. glomerata* subspecies (Table 5). Origin and distributional range information were from U.S. National Plant Germplasm System (https://npgsweb.ars-grin.gov), and the climate type were listed in accordance with previous report [50]. US National Plant Germplasm System (US NPGS) generously provide all the materials.

### 4.2. DNA Extraction

Each accession was represented by 6–10 plants, and fresh leaf tissues were collected from each plant and preserved in silica gel desiccant. Genomic DNA was extracted using a modified CTAB method [51]. The quality and concentration of each DNA sample were assessed by NanoVue Plus spectrophotometry (General Electric Company, England, UK) and electrophoresis on 1% agarose gel. Isolated genomic DNA was diluted to 10 ng/L and stored at −20 °C for use.

### 4.3. Primer Selection and PCR Amplification 

According to previous reports [52,53], a total of 100 *Dactylis* SSR primers have been described and 21 primers amplifying relatively more numbers and clear diversity bands were selected in our study (Table 6). PCR reactions were carried out in a volume of 15 μL containing 40 ng genomic DNA, 0.4 U Taq polymerase, 0.6 μM each primer (forward and reverse), and 7.5 μL Golden easy PCR mix (Tiangen Biotech, Beijing, China). PCR cycling parameters were carried out as described by Xie et al. [54].

Based on preliminary tests, 15 pairs of IT-ISJ primers were selected from among 204 combinations of 6 forward and 34 reverse primers (Table 7) published in Zheng et al. [37] PCR reactions were carried out in a volume of 15 μL containing 40 ng genomic DNA, 0.5 U Taq polymerase, 0.75 μM each primer, and 7.5 μL Golden easy PCR mix. PCR cycling parameters were carried out as described by Zheng et al. [37], with a slight adjustment as follows: initial denaturation at 94 °C for 5 min, 40 cycles of denaturing at 94 °C for 45 s, annealing at 50–55 °C for 45 s, and elongation at 72 °C for 1 min, a final elongation at 72 °C for 10 min, followed by preservation at 4 °C.

Amplified fragments were separated on 6% denatured polyacrylamide gels, with D2000 as a size marker. After electrophoresis, the gel was stained with AgNO_3_ solution and photographed by digital camera.

### 4.4. Data Analysis

All analyses were conducted by grouping SSR and IT-ISJ marker data together. Unambiguous amplified bands of each SSR and IT-ISJ marker were scored as 1 for presence and 0 for absence. The total number of bands (TNB), number of polymorphic bands (NPB), and percentage of polymorphic bands (PPB) were obtained from the gels. The polymorphism information content (PIC) of each SSR and IT-ISJ locus was estimated using the method of Nei [55] according to the formula: PIC*i* = 1 − ∑P*_ij_*^2^, where P*_ij_* is the frequency of the *j*th allele for *i*th locus, summed across all alleles of the locus. The average PIC of each primer pair was determined by PIC*a* = ∑PIC*i*/N, where N is the number of polymorphic bands per primer. Meanwhile, the efficiencies of SSR and IT-ISJ markers were assessed by marker index (MI), which includes the average band informativeness (Ibav) for the polymorphic markers and the effective multiplex ratio (EMR) [40]. Ibav was calculated with the formula: Ibav = 1/n∑1 − (2 × |0.5 − pi|), where “n” is the total number of amplification sites and pi represents the proportion of the ith amplification site. EMR is the average number of polymorphic bands [56]. 

Population genetic parameters including Nei’s gene diversity index (He) [55] and Shannon’s diversity index (I) [57] for each accession were estimated using POPGENE v.1.3.2 (University of Alberta, Edmonton， CA, USA) with a model for dominant markers and diploid individuals. Meanwhile, an analysis of molecular variance (AMOVA) was performed to determine the variance within and among the nine subspecies by AMOVA version 1.55 (University of Geneva, Geneva, Switzerland). For subspecies that included accessions from different countries, AMOVA was also performed to determine the variance within and among countries. Data input files for POPGENE and AMOVA were managed by the DCFA1.1 program written by Zhang [58]. Genetic similarities were calculated between pairs of plants using Dice coefficients [59] with the NTSYS-pc2.10 software package [60], and the average genetic similarities between pairs of plants in two species were regarded as genetic similarities between these two subspecies. We obtained the average genetic similarities between each pair of accession. A neighbor joining unweighted phylogenetic tree was constructed based on the Dice dissimilarity matrix between 196 individuals and 1000 bootstrap replicates were performed using the Darwin software package v5.0148 [61]. Population structure was also analyzed using STRUTURE software v.2.3.4 [62]. The analyses were performed under the admixture model and 10 independent runs for K values ranging from 1 to 20 were performed with a burn-in 50,000 and 100,000 iterations of Markov chain convergence. Furthermore, the most probable value of K was determined in accordance with Haidong Yan et al. [47].

## 5. Conclusions

In this study, we compared the efficiencies of SSR and IT-ISJ markers, revealing that SSRs exhibit higher efficiency. The 20 accessions of 9 subspecies from various countries were included in our study, exhibiting a high level of genetic diversity. Furthermore, the result of cluster analysis had a certain relationship with distribution ranges. Phylogenetic analysis and structure analysis indicated that genetic relationships among *Dactylis* subspecies may also be associated with their climate types. The materials from the Temperate type could be divided into individual Group C or Cluster 5, and the materials from the subtropical type together with some materials from the Mediterranean type formed one group (A), while the remaining materials of the Mediterranean were gathered into a different group (B). Furthermore, according to the origin of our materials, the materials from western and eastern Mediterranean could be divided into different groups (A and B).

## Figures and Tables

**Table 1:** Amplification results from SSR and IT-ISJ primer combinations.

Primer Code	TNB	NPB	PPB (%)	PIC	Primer Code	TNB	NPB	PPB (%)	PIC
FOG215	11	11	100	0.916	IT-ISJ01F03R	8	8	100	0.616
FOG238	9	9	100	0.768	IT-ISJ01F04R	8	8	100	0.874
FOG258	13	13	100	0.909	IT-ISJ01F05R	5	5	100	0.957
FOG296	6	6	100	0.869	IT-ISJ01F08R	6	6	100	0.710
FOG362	10	10	100	0.962	IT-ISJ03F41R	9	9	100	0.833
FOG402	8	8	100	0.861	IT-ISJ03F47R	7	7	100	0.741
FOG514	11	11	100	0.959	IT-ISJ03F59R	10	10	100	0.728
FOG515	10	10	100	0.940	IT-ISJ04F04R	6	6	100	0.626
FOG537	7	7	100	0.938	IT-ISJ05F20R	7	7	100	0.835
FOG591	10	10	100	0.916	IT-ISJ05F36R	3	3	100	0.924
FOG634	9	9	100	0.928	IT-ISJ06F50R	3	3	100	0.911
FOG655	8	8	100	0.910	IT-ISJ07F05R	7	7	100	0.814
FOG707	8	8	100	0.956	IT-ISJ07F11R	7	7	100	0.727
FOG112	10	10	100	0.905	IT-ISJ07F12R	8	8	100	0.852
FOG393	8	8	100	0.948	IT-ISJ07F15R	5	5	100	0.545
FOG455	10	10	100	0.872	Total	99			
FOG598	8	8	100	0.920	Mean	6.600			0.780
FOG624	9	9	100	0.871					
FOG824	9	9	100	0.875					
FOG831	13	13	100	0.952					
DGSSR14primer12	9	9	100	0.914					
Total	196								
Mean	9.333			0.909					

TNB = total number of bands; NPB = number of polymorphic bands; PPB = percentage of polymorphic bands; PIC = polymorphism information content.

**Table 2:** Efficiency parameters of SSR and IT-ISJ markers.

Items	SSR	IT-ISJ
No. of primers	21	15
No. of total loci	196	99
No. of average loci per primer	9.33	6.6
Percentage of polymorphic bands (PPB)	100	100
Average band informativeness (Ibav)	0.49	0.54
Effective multiplex ratio (EMR)	9.33	6.60
Marker index (MI)	4.58	3.54

**Table 3:** Genetic variation statistics and Shannon’s diversity estimation for all subspecies.

Subspecies	Na	Ne	He	*I*	Distribution of Genetic Diversity	
*D. glomerata* subsp*. santai*	1.898	1.409	0.257	0.401	*It*	0.448
*D. glomerata* subsp*. smithii*	1.871	1.372	0.235	0.370	*Is*	0.321
*D. glomerata* subsp*. woronowii*	1.807	1.344	0.217	0.340	*Is/It*	0.717
*D. glomerata* subsp*. lusitanica*	1.471	1.213	0.135	0.211	*S'*	0.283
*D. glomerata* subsp*. himalayensis*	1.556	1.255	0.159	0.248		
*D. glomerata.* subsp*. glomerata*	1.536	1.264	0.162	0.251		
*D. glomerata* subsp*. lobata*	1.881	1.368	0.232	0.366		
*D. glomerata* subsp*. hispanica*	1.993	1.420	0.269	0.425		
*D. glomerata* subsp*. marina*	1.519	1.307	0.184	0.276		
Mean	1.726	1.328	0.205	0.321		
Total	2.000	1.433	0.283	0.448		

Na = observed number of alleles; Ne = effective number of alleles; He = expected heterozygosity or Nei’s gene diversity; *I* = Shannon’s diversity index; *It* = total diversity; *Is* = intra-subspecies diversity; *Is/It* = proportion of intra-subspecies diversity; *S'* = proportion of inter-subspecies diversity.

**Table 4:** AMOVA of 20 subspecies and across countries where applicable.

Source of Variation	Sum of Squares	Variance Component	Total Variation (%)	Degree of Freedoms	*p*-Values
20 accessions					
Variance among accessions	2132.571	10.892	0.191	19	<0.001
Variance within accessions	8661.510	46.072	0.809	176	<0.001
*D. glomerata* subsp*. hispanica*					
Variance among countries	1384.837	11.434	0.108	6	<0.001
Variance within countries	6139.533	94.454	0.892	63	<0.001
*D. glomerata* subsp*. lobata*					
Variance among countries	165.050	8.970	0.165	2	<0.001
Variance within countries	1272.750	45.455	0.835	27	<0.001
*D. glomerata* subsp*. smithii*					
Variance among countries	144.800	10.340	0.200	2	<0.001
Variance within countries	745.200	41.400	0.800	17	<0.001
*D. glomerata* subsp*. woronowii*					
Variance among countries	141.300	10.508	0.225	2	<0.001
Variance within countries	652.000	36.222	0.775	17	<0.001

**Table 5:** Accession numbers, origin, distributional range, and climate type of materials.

Code	Name	Taxon	Origin and Locality	Distribution Range	Climate Type
1	PI237605	*D. glomerata* subsp*. santai*	Mountain Tessala, near Sidi Abbes, Algeria	Unknown	Mediterranean
2	PI368880	*D. glomerata* subsp. *santai*	Mountain Tessala, north of Sidi Abbes, Algeria	Unknown	Mediterranean
3	PI441034	*D. glomerata* subsp. *smithii*	United Kingdom (Portugal-madeira Islands; spain-Canary Islands)	Africa	Subtropical
4	PI237607	*D. glomerata* subsp*. smithii*	Tenerife, Canary Islands, Spain	Africa	Subtropical
5	PI538922	*D. glomerata* subsp. *woronowii*	Russian Federation, Leningrad	Asia-Temperate	Mediterranean
6	PI237610	*D. glomerata* subsp. *woronowii*	Tehran, Iran	Asia-Temperate	Mediterranean
7	PI237602	*D. glomerata* subsp. *lusitanica*	Near Sintra, Algueirao, Portugal	Europe	Mediterranean
8	PI295271	*D. glomerata* subsp. *himalayensis*	India	Asia-Tropical	Temperate
9	PI538920	*D. glomerata* subsp. *glomerata*	Russian Federation	Unknown	Temperate
10	PI316209	*D. glomerata* subsp. *lobata*	Bulgaria	Europe	Temperate
11	PI372621	*D. glomerata* subsp. *lobata*	Bremen, Germany	Europe	Temperate
12	PI283242	*D. glomerata* subsp. *lobata*	Germany	Europe	Temperate
13	PI231517	*D. glomerata* subsp. *hispanica*	Midelt, Morocco	Africa	Mediterranean
14	PI265568	*D. glomerata* subsp. *hispanica*	Spain	Europe (Canry Islands) Africa (Baleares)	Mediterranean
15	PI265567	*D. glomerata* subsp. *hispanica*	France	Europe	Mediterranean
16	PI224599	*D. glomerata* subsp. *hispanica*	Ruhama, Israel	Unknown	Mediterranean
17	PI277836	*D. glomerata* subsp. *hispanica*	Turkey	Asia-Temperate	Mediterranean
18	PI231550	*D. glomerata* subsp. *hispanica*	Agrinion, Greece	Europe	Mediterranean
19	PI231541	*D. glomerata* subsp. *hispanica*	Nazare, Portugal	Europe	Mediterranean
20	PI577065	*D. glomerata* subsp. *marina*	Sao Vivent, Portugal	Europe	Mediterranean

**Table 6:** SSR primer sequences.

Primer Name	Forward Primer (5′→3′)	Reverse Primer (5′→3′)
FOG215	CAGTGACTACCGTCGTTACTC	TTGCTGCAAGGAAAATTC
FOG238	GTCACCTAAGCCATAGCAAG	ACTTCTGTGTTGGTACCGAC
FOG258	GCAGTATGGTGCTCTCTCTT	CACTCGTTCAGATCGTCC
FOG296	ATGGAAGTTTCCTGGAATG	GAAGCAGAGTAGAGCCCAC
FOG362	ATTGCATGGTTCTGCACT	GTGAGTATGCGTGTTTGCTA
FOG402	TCCTTATGAAATGAATGAATGA	AAGAACTGGACATATACTTGGG
FOG514	CTGATTCGATATGAATGCTTC	ACATGATTGAGAAACGGAAC
FOG515	GATGAAGGAACTGCTGGAT	ACACCAGACCCTAAACAGC
FOG537	TAAATCTTGCACTTATCTGTGC	AACTGTACTCTCTCACACCCTC
FOG591	CTCATGCAAGATATGGCAC	AAGATCAGGTTTGAACCTCTC
FOG634	GTGCGTCTTTTAAATGGTATG	GAGCCTCCCTAACCCTAGTA
FOG655	GAGATGAGCCATGATTCATT	GTGCTTGCTTGATTCACC
FOG707	CGCTAGAACCTCCCTACAC	CAAAAGATCTTCAACGCTG
FOG112	TAAGAATCGATCCTCCCG	ACCTTCTTCCACTCCGTC
FOG393	GGGGAGGTACCCACTTCT	TTACCCAATCTAAGATCTTTGC
FOG455	GTGAGGTTGTGGAAAGTGAC	CAAAACTAACGCCGTTAACT
FOG598	GCCAGGTCAGTCACTCAC	AGAAAATTCCCCAACAGC
FOG624	CATCAGGGTAGTATCCCGTA	AGTTCCTTCTCCTCTGCTTT
FOG824	TAGTGGAATGTCAAGAAAATGA	AAAATGTCCTTGTTCCCAG
FOG831	TAAAGCATATGCAACAATGC	TGCTAAAGCCTTTTACAGCT
DGSSR14 primer12	AGGCCTTCTTGCACTGGTAC	GAGTTCACTGAGGCCGAGAG

**Table 7:** IT-ISJ primer sequences.

Primer Name	5′→3′ Sequence	Primer Name	5′→3′ Sequence
Forward primer		Reverse primer	
IT-ISJ01F	GCATGCCAGGTAAGTAAA	IT-ISJ24R	GGAATTCCACCTGCACCT
IT-ISJ03F	GCATGCCAGGTAAGTAAG	IT-ISJ26R	GGAATTCCACCTGCACGC
IT-ISJ04F	GCATGCCAGGTAAGTAAT	IT-ISJ28R	GGAATTCCACCTGCACGT
IT-ISJ05F	GCATGCCAGGTAAGTACA	IT-ISJ29R	GGAATTCCACCTGCACTA
IT-ISJ06F	GCATGCCAGGTAAGTACC	IT-ISJ32R	GGAATTCCACCTGCACTT
IT-ISJ07F	GCATGCCAGGTAAGTACG	IT-ISJ36R	GGAATTCCACCTGCAGAT
Reverse primer		IT-ISJ38R	GGAATTCCACCTGCAGCC
IT-ISJ01R	GGAATTCCACCTGCAAAA	IT-ISJ40R	GGAATTCCACCTGCAGCT
IT-ISJ03R	GGAATTCCACCTGCAAAG	IT-ISJ41R	GGAATTCCACCTGCAGGA
IT-ISJ04R	GGAATTCCACCTGCAAAT	IT-ISJ42R	GGAATTCCACCTGCAGGC
IT-ISJ05R	GGAATTCCACCTGCAACA	IT-ISJ44R	GGAATTCCACCTGCAGGT
IT-ISJ06R	GGAATTCCACCTGCAACC	IT-ISJ47R	GGAATTCCACCTGCAGTG
IT-ISJ07R	GGAATTCCACCTGCAACG	IT-ISJ50R	GGAATTCCACCTGCATAC
IT-ISJ08R	GGAATTCCACCTGCAACT	IT-ISJ51R	GGAATTCCACCTGCATAG
IT-ISJ11R	GGAATTCCACCTGCAAGG	IT-ISJ52R	GGAATTCCACCTGCATAT
IT-ISJ12R	GGAATTCCACCTGCAAGT	IT-ISJ54R	GGAATTCCACCTGCATCC
IT-ISJ13R	GGAATTCCACCTGCAATA	IT-ISJ58R	GGAATTCCACCTGCATGC
IT-ISJ15R	GGAATTCCACCTGCAATG	IT-ISJ59R	GGAATTCCACCTGCATGG
IT-ISJ20R	GGAATTCCACCTGCACAT	IT-ISJ63R	GGAATTCCACCTGCATTG
IT-ISJ22R	GGAATTCCACCTGCACCC	IT-ISJ64R	GGAATTCCACCTGCATTT
IT-ISJ23R	GGAATTCCACCTGCACCG		

## Supplementary Materials

Supplementary materials can be accessed at: http://www.mdpi.com/1420-3049/21/11/1459/s1.
