# Supplementary material for: Phylogenetic and Diversity Analysis of Dactylis glomerata Subspecies Using SSR and IT-ISJ Markers
Source: Molecules. 2016 Oct 31;21(11):1459. doi: 10.3390/molecules21111459 (PMC6272990; doi:10.3390/molecules21111459)
Supplement: Supplementary file 1 [file molecules-21-01459-s001.pdf]

Supplementary Materials: Phylogenetic and Diversity Analysis of *Dactylis glomerata* Subspecies Using SSR and IT-ISJ Markers

Defei Yan, Xinxin Zhao, Yajuan Cheng, Xiao Ma, Linkai Huang and Xinquan Zhang

Table 1. Genetic variation statistics and Shannon's diversity estimation for all accessions.

Subspecies	Na	Ne	He	*I*	Distribution of Genetic Diversity	
D. glomerata subsp. *santai* A1	1.783	1.420	0.252	*0.384*	*It*	0.448
D. glomerata subsp. *santai* A2	1.746	1.328	0.207	*0.326*	*Ia*	0.302
D. glomerata subsp. *smithii* S	1.722	1.341	0.210	0.327	*Ia/It*	0.671
D. glomerata subsp. *smithii* UK	1.709	1.330	0.205	0.320	*P′*	0.329
D. glomerata subsp. *woronowii* I	1.627	1.299	0.184	0.285		
D. glomerata subsp. *woronowii* R	1.600	1.305	0.187	0.288		
D. glomerata subsp. *lusitanica*	1.471	1.213	0.135	0.211		
D. glomerata subsp. *himalayensis*	1.556	1.255	0.159	0.248		
D. glomerata subsp. *glomerata*	1.536	1.264	0.162	0.251		
D. glomerata subsp. *lobata* B	1.654	1.311	0.192	0.298		
D. glomerata subsp. *lobata* G1	1.617	1.310	0.190	0.292		
D. glomerata subsp. *lobata* G2	1.620	1.295	0.184	0.286		
D. glomerata subsp. *hispanica* F	1.736	1.337	0.210	0.329		
D. glomerata subsp. *hispanica* G	1.695	1.331	0.206	0.320		
D. glomerata subsp. *hispanica* I	1.712	1.346	0.214	0.331		
D. glomerata subsp. *hispanica* M	1.532	1.269	0.167	0.258		
D. glomerata subsp. *hispanica* P	1.661	1.323	0.201	0.310		
D. glomerata subsp. *hispanica* S	1.807	1.388	0.239	0.370		
D. glomerata subsp. *hispanica* T	1.722	1.346	0.216	0.338		
D. glomerata subsp. *marina*	1.519	1.307	0.184	0.276		
Mean	1.651	1.316	0.195	0.302		
Total	2.000	1.433	0.283	0.448		

Na = observed number of alleles; Ne = effective number of alleles; He = expected heterozygosity or Nei's gene diversity; *I* = Shannon's diversity index; *It* = total diversity; *Ia* = intra-accession diversity; *Ia/It* = proportion of intra-accession diversity; *P′* = proportion of inter-accession diversity
